# Spatial forecasting of seismicity provided from Earth observation by space satellite technology

**DOI:** 10.1038/s41598-020-66478-9

**Published:** 2020-06-16

**Authors:** Gregorio Farolfi, Derek Keir, Giacomo Corti, Nicola Casagli

**Affiliations:** 1 Italian Military Geographic Institute, Firenze, Italy; 2 Department of Earth Sciences, University of Firenze, Firenze, Italy; 3 School of Ocean and Earth Science, University of Southampton, Southampton, United Kingdom; 4 Institute of Geosciences and Geo Resources, National Research Council of Italy, Firenze, Italy; 5 National Institute of Oceanography and Applied Geophysics - OGS, Trieste, Italy

**Keywords:** Geophysics, Seismology, Tectonics, Environmental sciences, Natural hazards, Solid Earth sciences, Applied physics

## Abstract

Understanding the controls on the distribution and magnitude of earthquakes is required for effective earthquake forecasting. We present a study that demonstrates that the distribution and size of earthquakes in Italy correlates with the steady state rate at which the Earth’s crust moves. We use a new high-resolution horizontal strain rate (S) field determined from a very dense velocity field derived from the combination of Global Navigation Satellite System (GNSS) and satellite radar interferometry from two decades of observations. Through a statistical approach we study the correlation between the S and the magnitude of M ≥ 2.5 earthquakes that occurred in the same period of satellite observations. We found that the probability of earthquakes occurring is linked to S by a linear correlation, and more specifically the probability that a strong seismic event occurs doubles with the doubling of S. It also means that lower horizontal strain rate zone can have as large earthquakes as high horizontal strain rate zones, just with a reduced probability. The work demonstrates an independent and quantitative tool to spatially forecast seismicity.

## Introduction

At seismically active plate boundaries, forecasting the distribution and sizes of earthquakes is fundamental for seismic hazard assessment. Approaches for seismic hazard estimation were traditionally based on forecasting the probability of future earthquakes from the statistical analysis of historical and instrumental earthquake catalogues^1^. However, this approach is flawed in most regions of continental deformation since the recurrence interval for earthquakes exceeds the length of the historical seismic record^2^. Instead, an alternative and independent approach in forecasting earthquake activity in regions that are deforming relatively fast involves using geodetic measurements of interseismic strain-rate as a proxy for future seismicity^3^. The theory of elastic rebound forms the basis of this method by considering that the elastic potential energy budget in the seismogenic crust is released by seismic events. With this assumption, the rate of elastic strain accumulation should correlate with the rate of strain release by earthquakes. Such an approach has successfully been implemented qualitatively for the San Andreas Fault system of California^4^ and North Anatolian system of Turkey^3^. Since regions of high strain rate should theoretically rupture more frequently, a quantitative correlation between increased frequency of earthquakes of any magnitude and strain rate should exist even for an earthquake catalogue that does not sample the full seismic cycle.

**Figure 1:**
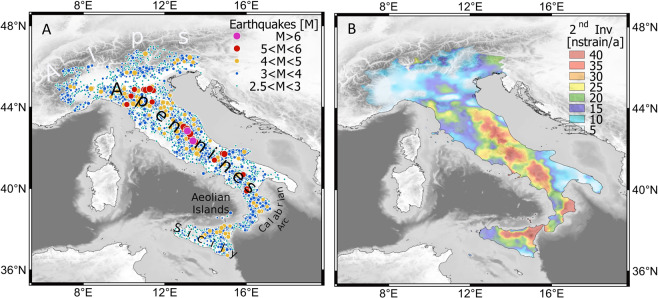
(**A**) Map of the distribution and magnitude of the 24555 M $\ge 2.5$ earthquakes that occurred in the Italian crust from January 1990 to December 2017. The earthquakes are represented by points with size increasing with magnitude. Seismicity is mainly distributed along the Apennines, the Calabrian Arc and sporadically in the Alps. (**B**) Map of the Second Invariant of the horizontal strain rate. Strain rate present high values long the Apennines, in the South-Eastern Tyrrhenian Sea including Aeolian Islands and north-eastern of Sicily and Calabrian Arc. Maps were created by Quantum GIS open source software (www.qgis.org) and using shaded relief map ETOPO1 Global Relief Model.

We test whether the rate of seismicity can be forecast from interseismic strain-rate by using the distribution and magnitude of crustal earthquakes reported in the Istituto Nazionale Geofisica e Vulcanologia (INGV) earthquake catalogue^5^ during 1990 to 2017 complete above magnitude (M) 2.5 (Fig. 1). We also compute a new and high-resolution strain rate map of Italy using geodetic data from the same time period. To do this we compute ground velocities using Global Navigation satellite Systems (GNSS) permanent stations and campaigns^6–8^, and integrate with ground velocities from Persistent Scatterers (PS) Interferometric Synthetic Aperture Radar (InSAR)^9^. The merging of GNSS and PSInSAR^10–13^ is used to produce an improved map of the horizontal strain rate (S), that quantifies the change in horizontal strain (deformation) of the Earth’s crust. The excellent seismicity catalogue combined with the well constrained and relatively fast strain rates of Italy make our data ideal to quantitatively test the correlation between position and size of earthquakes and S.

Tectonically, Italy is on the plate boundaries between the colliding African and Eurasian plates, that has been forming the Alpine and Apennine belts since around 90 Myrs ago^14^. Seismological data, recent geodetic studies and analysis of active faults reveal spatial variations of the deformation style and kinematics^15^. Overall, the Apennines and the Tyrrhenian side are undergoing a NE-directed extension, accommodated by moderate to large normal faulting earthquakes^16–19^. Conversely, the external front of the Apennines, the Po Plain and the southern Alpine front are undergoing active compression, accommodated by moderate magnitude reverse faulting seismicity (Fig. 1A). However, extension and compression may locally coexist and affect different crustal levels where generally extension is shallower than compression^20^. Overall, the region is characterized by moderate seismicity^21^, mainly distributed along the Apennines, the Calabrian Arc and sporadically in the Alps (Fig. 1A).

## Results

### Seismicity catalogue and strain rate computation

We used seismicity in Italy from January 1990 to December 2017 reported by the INGV catalogue, which is complete above M 2.5. We first isolated 24255 earthquake of magnitude M ≥ 2.5 and those that occurred in the crust using the Moho depth map reported by Labrousse^22^. Earthquakes typically have horizontal uncertainties in their position of less than 5 km. The size, low magnitude of completeness, and well constrained earthquake locations makes the INGV it one of the most comprehensive earthquake catalogues for a tectonically active region on Earth.

The map of strain rates of Italy was derived from a geodynamic velocity field of Italy calculated over the period 1994 to 2014. To compute the velocity field, we integrated the velocities of 621 GNSS sites with 25 million PS data points^13^. The resultant single and very dense velocity field is then gridded at 20 × 20 km and used to compute the two-dimensional velocity gradient tensor (Fig. 1B), from which the second invariant of the horizontal strain rate is derived (see methods section for the full description of how S maps were derived).

With our earthquake catalogue and S maps we use a Bayesian, or conditional approach, to determine the probability function for the occurrence of an earthquake of a given magnitude within a given S. From the analysis that we describe below we are able to determine that the seismicity rate increase with S by a linear trend. We show that the probability of an earthquake of any magnitude is highest at high strain rates following the Gutenberg-Richter law^23,24^.

### Probability density functions of earthquake magnitude and horizontal strain rate

**Figure 2:**
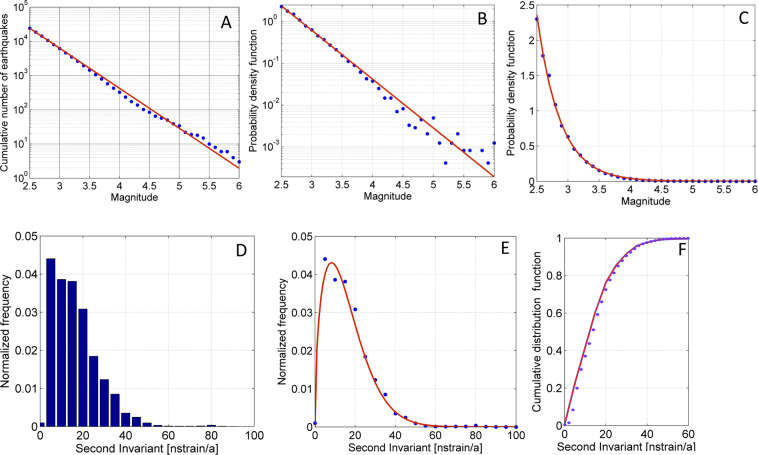
(**A**) Cumulative number of earthquakes that occurred from January 1990 to December 2017 in a logarithmic scale. Coefficients are a = 7.31, b = 1.17. (**B**,**C**) show the probability density functions $f(M\ge 2.5)$ in a logarithmic and liner scale respectively with earthquakes cumulated in classes of 0.1 magnitude units. The red line represents the fitted curve from the Gutenberg-Richter law. Normalized histogram (**D**) and the Weibull probability density function (**E**) defined for positive strain rates. Cumulative distribution function (**F**) of strain rates in classes of 5 nstrain/a determined for the study area. Graphs were developed by GNU Octave Scientific Programming Language (https://www.gnu.org/software/octave/).

The first step in our analysis is to compute the probability density function of magnitudes in the earthquake catalog from the Gutenberg-Richter distribution;

1 $$N(M)={10}^{a-bM}$$

where N is the cumulative number of earthquakes (Fig. 2A) with magnitude (M) higher or equal to the magnitude of completeness (${m}_{c}$) which is the lowest magnitude at which the seismic-event catalogue is complete. The parameters *a* and *b* are respectively the earthquake productivity of a volume and the relative size coefficient distribution^25^ (Fig. 2C). From the Eq. 1, it follows that the probability density function $f(M)$ for events with $M\ge {m}_{c}$^26^ is:

2 $$f(M)=\beta {e}^{-\beta (M-{m}_{c})},\,M\ge {m}_{c},\,{\rm{with}}\,\beta =b.ln(10)=2.69\,{\rm{and}}\,{m}_{c}=2.5.$$

The second stage of the analysis was to determine the distribution of S. The distribution of horizontal strain rates of the study area *d* $(S)$ was determined from the normalized histogram of S (Fig. 2D,E). We hypothesise that S follows the Weibull distribution (Fig. 2F):

3 $$d(S)=\lambda \,\mu \,{s}^{\mu -1}{e}^{-\lambda {s}^{\mu }},$$

where *λ* = 0.01426 and *µ* = 1.492 are the parameters of the maximum likelihood estimation. Determination of *d* $(S)$ permits identification of the distribution of S, as well as the upper limit of S that correspond to the value of 0.99 of its cumulative distribution function.

### Correlation between seismicity and horizontal strain rate

**Figure 3:**
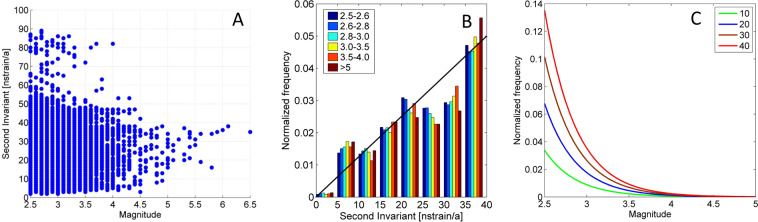
(**A**) Second invariant strain rate plotted against earthquake size. (**B**) is the normalized values of the density function of S determined for bins of 5 nstrain/a represented for classes of magnitudes. The black line represents the hypothesized probability density function of S. It shows a linear trend for positive values until 40 nstrain/a, corresponding to the limit of significance (96% of the dataset). (**C**) is the relative frequencies of S represented in class of magnitude values calculated for intervals of 10 nstrain/a in a linear scale for events with $M\ge 2.5$. Graphs were developed by GNU Octave Scientific Programming Language (https://www.gnu.org/software/octave/).

*To correlate seismicity and horizontal strain rate (S) we associated to each earthquake the strain rate of the 20* × *20 km grid cell in which the earthquakes occurred. We therefore obtained a dataset in which for each earthquake we know the earthquake* magnitude and strain rate. The magnitude of earthquakes and the S are plotted against each other in Fig. 3A. Since we had to determine the mutual condition probability $f(S|M)$, which is the probability function of the strain rate S given an earthquake of a particular magnitude, we calculated the distributions of $f(S|M)$ for different classes of magnitudes and display the results in Fig. 3B. The results show that the distribution of strain rates is stochastically independent of the magnitude of events. This means that earthquakes grouped into classes of magnitude have the same probability density as a function of the strain rate $S$. In addition, the probability of earthquakes occurring increases only with the strain rate $S$ and we can therefore write $f(S|M)$ = $f(S|eqk)$. We interpret that $f(S|eqk)$ is a linear trend until the maximum value of the strain rate ${s}_{max}$ of the study area which is ~40 nstrain/a (Fig. 3B):

4 $$f(S|eqk)=\frac{2\,}{{s}_{max}^{2}}S,\,{\rm{for}}\,{\rm{S}}\,\in [0,{s}_{max}]$$

that satisfied the normalization condition:

5 $${\int }_{0}^{{s}_{max}}f(S|eqk)dS=1$$

Finally, we determined the probability that a seismic event of magnitude *M* occurs in an area with a given S as a combination of independent events:

6 $$f(M\cap S)=f(M).f(S|eqk)$$

Replacing equations Eq. 2 and Eq. 4 in Eq. 6:

7 $$f(M\cap S)=\frac{2\,}{{s}_{max}^{2}}S\beta {e}^{-\beta (M-{m}_{c})}=2.82\,S{e}^{-\beta M},\,M\ge {m}_{c}$$

The resultant Eq. 7 preserves the Gutenberg-Richter law for the magnitude distribution of earthquakes. The results from applying Eq. 7 shows that the probability of earthquakes of any magnitude is linearly dependent on the strain rate (Fig. 3C).

## Discussion

**Figure 4:**
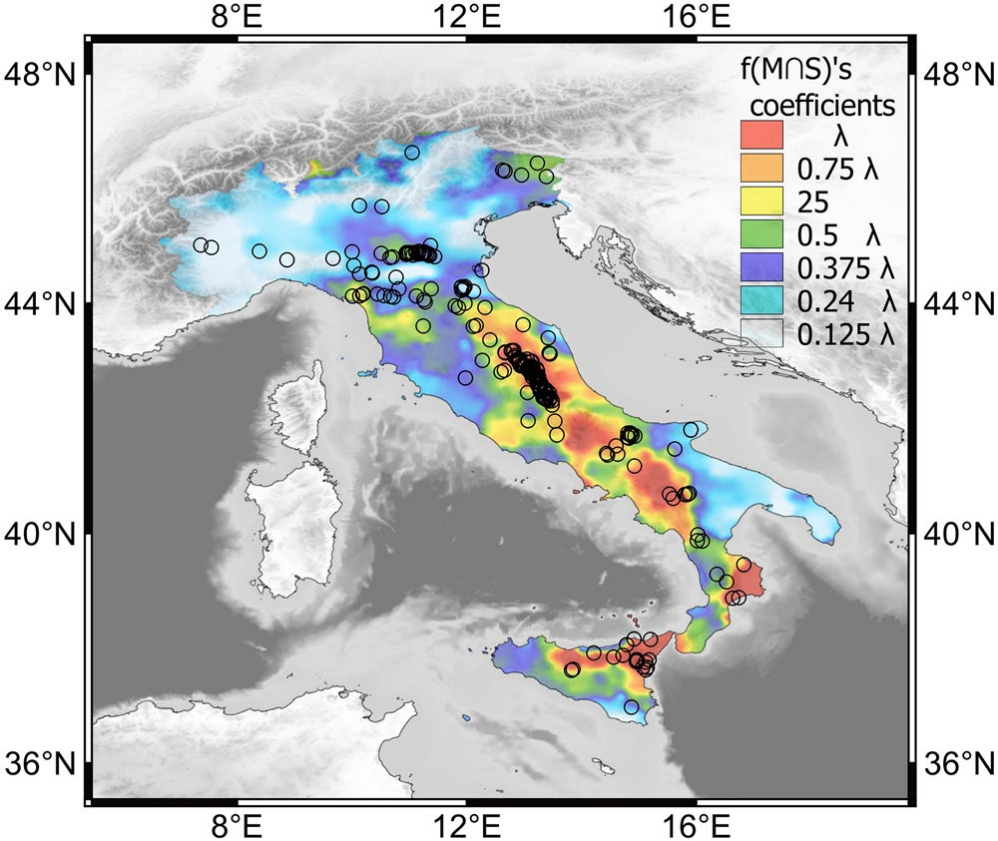
Map of $f(M\cap S) \acute{} s$ coefficient ${\rm{\lambda }}=\frac{2\,}{{s}_{max}^{2}}\,S\,\beta {e}^{\beta {m}_{c}}$ with earthquakes that occurred from 1990 to 2017 with $\,M\ge 4$. Map was created by Quantum GIS open source software (www.qgis.org) and using shaded relief map ETOPO1 Global Relief Model.

The linear dependency that links the occurrence of earthquakes of a particular magnitude with the increase in strain rates shows that regions of high inter-seismic strain rate are more likely to be seismically active across all earthquake magnitudes (Fig. 3A). This is consistent with the hypothesis that earthquakes are the sudden release of accumulated elastic strain. Our study is the first to statistically show a positive link between inter-seismic strain rate and the occurrence of earthquakes across a range of earthquake magnitudes. The relationship between strain rates and seismicity is also visually clear in map view. In Fig. 4 we plot the map of $f(M{\cap }^{}S) \acute{} s$ coefficient $\lambda =\frac{2\,}{{s}_{max}^{2}}\,S\,\beta {e}^{\beta {m}_{c}}$ with seismicity from the INGV catalogue, and the distribution of the larger earthquakes mainly occur where strain rates are highest.

Our work advances efforts to link seismic activity to interseismic strain rates. Previous studies from the San Andreas Fault in California have demonstrated that regions of high strain rate correlated with the occurrence of relatively large (M 6.5) earthquakes but with lower magnitude events showing a more complicated pattern^4^. Our results also differ from a previous analysis of the link between strain rates and seismicity in Italy^27^, which showed a statistical link between the occurrence of the largest magnitude earthquakes in Italy with low to moderate strain rates. Their study suggest that regions of locked faults have relatively lower inter-seismic strain rates but rupture in relatively larger events. In contrast to both these studies, we demonstrate that the probability of an earthquake occurring of any magnitude is highest in regions of elevated strain rate. Therefore, the results support the simple hypothesis that fault zones follow simple elastic rebound theory and exhibit Gutenberg-Richter behavior.

## Conclusions

We use a very dense velocity field derived by the integration of GNSS and InSAR over 20 years, and the INGV earthquake catalogue complete above magnitude 2.5, and demonstrate the relationship linking earthquake magnitude with horizontal geodetic strain rates. We found the probability of seismic events at all magnitudes linearly increases with the strain rate. Our approach quantitatively demonstrates that measuring the rate of inter-seismic strain accumulation is an effective method for forecasting the distribution and magnitude of earthquakes.

## Methods

### Geodetic velocity maps and strain rate computation

**Figure 5:**
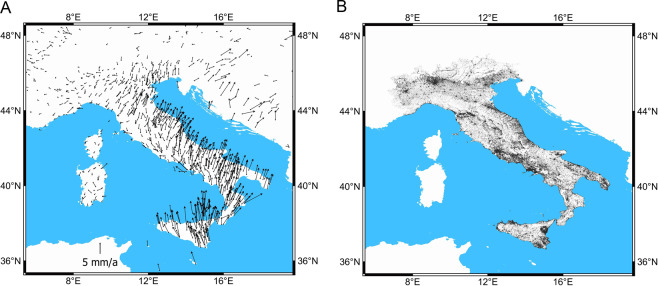
(**A**) is horizontal velocities of GNSS permanent stations aligned with respect to the Eurasian reference frame (ETRS89). (**B**) is the distribution of the 25 million PS with accurate and linear velocities recorded in the Italian peninsula. Maps were created by Quantum GIS open source software (www.qgis.org).

The map of the strain rate of Italy was derived from a geodynamic velocity field of Italy calculated over the period 1994 to 2014 by the integration of the velocities of 621 GNSS sites (Fig. 5A) with 25 million of PS^6^ (Fig. 5B). PSInSAR is a technique applied to satellite SAR images that produce persistent scatterers (PS), which are sparse ground point-wise radar benchmarks characterized by long-term stability of the electromagnetic backscattered signal and high reflectivity^28–30^. This accurate and very dense velocity field is the base for the determination of a correct geodetic strain field. Full details of the geodetic dataset are reported in Farolfi *et al*.^13^.

The two-dimensional velocity gradient tensor is calculated by applying the infinitesimal strain approach^31,32^. Due to the fine scale detail of horizontal velocity, and to reduce the size of the large dataset, we have mapped the velocity field and strain tensor with a grid of 20 km × 20 km. The known horizontal incremental velocity vector ${V}_{i}$ of the $i$ vertex polygon is defined as:

8 $${V}_{i}={A}_{i}+\frac{\partial {V}_{i}}{\partial {x}_{j}}{x}_{j}={A}_{i}+{t}_{ij}{x}_{j}$$

where ${A}_{i}\,$is the unknown velocity at the origin of the coordinate system, ${x}_{j}\,$ is the position of the station, and ${t}_{ij}$ is the displacement gradient tensor. Tensor theory states that any second-rank tensor can be separated into a symmetric and an anti-symmetric tensor then ${t}_{ij}$ can be additively decomposed asfollows:

9 $${t}_{ij}=\frac{({t}_{ij}+\,{t}_{ji})}{2}+\frac{({t}_{ij}-{t}_{ji})}{2}={e}_{ij}+{\omega }_{ij}$$

**Figure 6:**
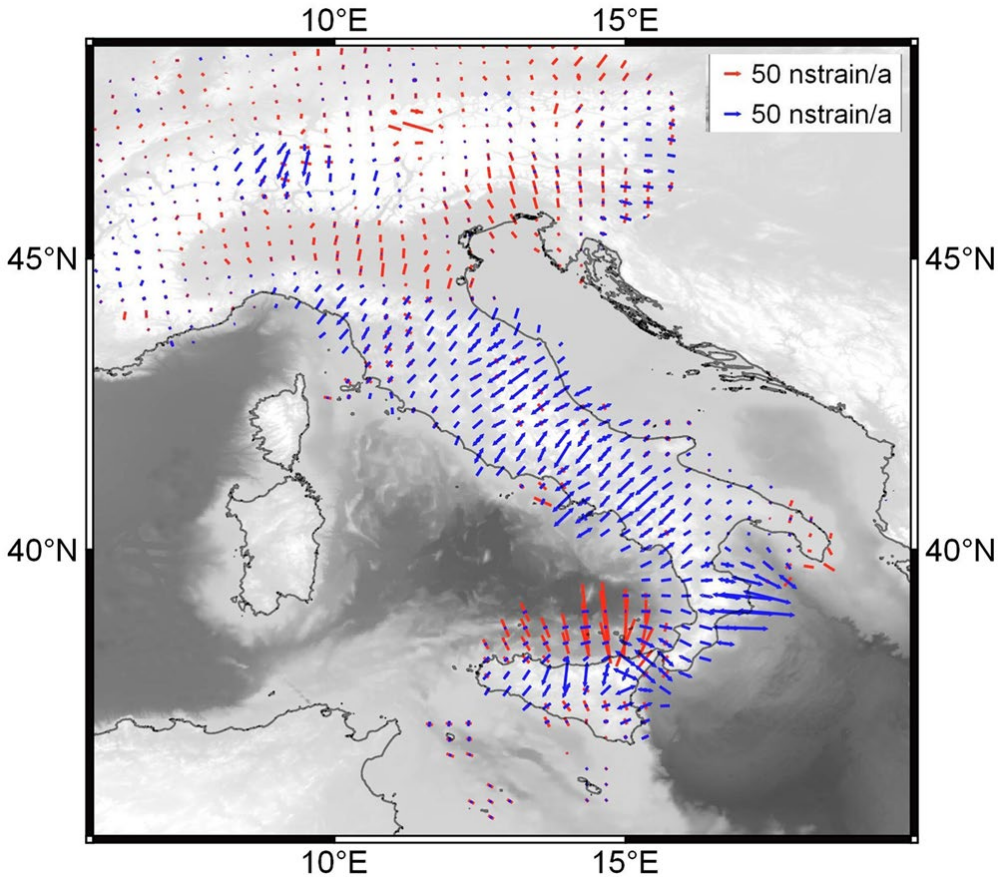
Principal horizontal strain rate axes computed with a distance weighted algorithm with a constant scale factor of 20 km. Lengthening are represented by symmetric blue arrows pointing outward and shortening are represented by symmetric red arrows pointing inward. Interpolated strain rates are shown over the sea. Map was created by Quantum GIS open source software (www.qgis.org) and using shaded relief map ETOPO1 Global Relief Model.

For infinitesimal strain rates, the symmetric and anti-symmetric parts can be associated with the infinitesimal strain ${e}_{ij}\,$and rotation ${\omega }_{ij}$ tensors. Principal strains (Fig. 6) were computed as:

10 $${e}_{1},\,{e}_{2}=\frac{1}{2}({e}_{ii}+{e}_{jj})\pm \frac{1}{2}\sqrt{{({e}_{ii}-{e}_{jj})}^{2}+4{e}_{ij}^{2}}$$

and the horizontal Second Invariant of the strain rate (SR) tensor has been evaluated as the scalars and represented in Fig. 2B:

11 $$SR=\sqrt[2]{{{e}_{1}}^{2}+{{e}_{2}}^{2}}$$
